# Own the turf or lose it: Ultrasound‐guided procedures and the future of shoulder repair

**DOI:** 10.1002/ksa.70405

**Published:** 2026-04-14

**Authors:** Alexandre Lädermann, Danilo Vukanic, Giuseppe Milano

**Affiliations:** ^1^ Division of Orthopaedics and Trauma Surgery Hôpital de La Tour Meyrin Switzerland; ^2^ Division of Orthopaedics and Trauma Surgery, Department of Surgery Geneva University Hospitals Geneva Switzerland; ^3^ Faculty of Medicine University of Geneva Geneva Switzerland; ^4^ FORE (Foundation for Research and Teaching in Orthopedics, Sports Medicine, Trauma, and Imaging in the Musculoskeletal System) Meyrin Switzerland; ^5^ Department of Trauma and Orthopaedics Wythenshawe Hospital, Manchester University NHS Foundation Trust Manchester UK; ^6^ Department of Medical and Surgical Specialties, Radiological Sciences, and Public Health University of Brescia Brescia Italy; ^7^ Department of Bone and Joint Surgery ASST Spedali Civili Brescia Italy

**Keywords:** minimal invasive surgery, office‐based procedures, percutaneous repair, rotator cuff disease, shoulder, ultrasound‐guided intervention, value‐based care

## Abstract

To highlight why the current arthroscopy‐centred paradigm may be insufficient for rotator cuff disease and to propose a surgeon‐led transition towards office‐based, ultrasound‐guided ‘micro’ interventions. The central question is whether earlier, less invasive image‐guided procedures can preserve tissue biology and improve value while maintaining patient safety. This commentary synthesises five decades of arthroscopic shoulder surgery principles with contemporary rotator cuff care pathways and the rapid expansion of musculoskeletal ultrasound outside the operating room. Emerging enabling technologies, including navigation, dedicated micro‐instruments, and standardised procedural workflows, are considered to outline a plausible near‐term shift in first‐line procedural care. Arthroscopy has transformed shoulder surgery, yet its foundational approach has changed little: fluid distension for visualisation, tissue ablation to create a view, and dependence on costly operating‐room infrastructure. In rotator cuff disease, clinical practice often tolerates prolonged delay, during which imaging, injections, and adjunctive therapies repeatedly consume resources while tendon and muscle degeneration progress. This delay can culminate in complex salvage procedures once reparative biology is compromised. In parallel, ultrasound has become ubiquitous and increasingly capable of supporting interventional solutions beyond diagnosis and injections. As barriers to adoption decrease, first‐line procedures may move upstream, potentially shifting the scope of practice away from surgeons unless they actively shape standards and evidence. The appropriate response is not territorialism but leadership. Shoulder surgeons should treat ultrasound literacy as foundational, adopt a deliberate micro‐interventional mindset, develop and validate safety‐first workflows, and generate comparative evidence that patients and health systems require. If surgeons lead this transition, earlier intervention may preserve biology and value; if not, the next paradigm will be defined elsewhere.

Half a century of arthroscopy: a revolution that became an evolution

Arthroscopy transformed orthopaedics and is one of the most important technological shifts our speciality has experienced in the modern era [3]. Early in the development of shoulder arthroscopy, enthusiasm was far from universal. Real scepticism existed, sometimes explicit, and often rooted in a reasonable concern: new technology can create new complications, new costs and new illusions of precision. The now‐famous quip attributed to Rockwood Jr., calling the arthroscope ‘the instrument of the devil’ [18], captured a broader sentiment among a generation trained in open anatomy and tactile certainty.

In several environments, adoption was slower than it should have been. In some countries and settings, non‐surgical clinicians and adjacent specialities, such as rheumatologists, explored arthroscopic approaches. Meanwhile, many orthopaedic surgeons hesitated, debated indications or waited for evidence that, in retrospect, was predictable. But that institutional delay mattered; disruptive technology does not wait for professional comfort. It created a window in which others could credibly claim competence and legitimacy. In France, for example, orthopaedic surgeons reportedly needed a decade or more to reassert leadership in arthroscopy after early adoption by rheumatologists [4, 6]. And, once again, those who move first may define the rules.

Yet, half a century later, it is striking how much of the foundational paradigm remains unchanged. We continue to ‘fill with water’ and to ‘coagulate perfusing vessels’ just to see. We remain reliant on extensive, costly infrastructure: operating rooms, towers, optics, pumps, cannulas, tubing, shavers, radiofrequency devices, etc. The choreography has improved, the optics have sharpened, anchors and sutures have evolved, and the learning curves are better supported. Still, the central concept is remarkably familiar to surgeons who trained decades apart. Although I (A.L.) understand subscapularis pathology better today, I still repair it largely as I learned 18 years ago—perhaps still waiting for the next revolution.

The paradox of rotator cuff care: we often wait until biology is lost

Few areas illustrate our current inertia better than rotator cuff disease. Part of this inertia is driven by how certain literature has been interpreted to suggest that a large proportion of rotator cuff tears are ‘asymptomatic’, fostering the view that tears are para‐physiological and simply part of normal ageing—something we should resign ourselves to accepting as a sign of time. That framing can implicitly legitimise a stepwise strategy in which repair is reserved only for truly symptomatic tears, or for those that remain symptomatic after repeated courses of conservative treatment, in the name of a supposedly appropriate therapeutic approach that balances costs and surgical effectiveness—even though that balance, and the ‘right’ threshold for delaying or avoiding repair, has not been properly verified or demonstrated. In many patients with small or moderate tears, we tolerate prolonged symptom‐guided surveillance while the structural lesion can progress [2]. During that interval, tendons may retract, muscles atrophy and fatty infiltration develops [23], changes that correlate with inferior healing potential [9] and more complex decision‐making later [13]. Preclinical work has shown that delaying tendon‐to‐bone repair can reduce biomechanical properties and is associated with bone loss at the repair site [8]. Clinical series also support the intuitive point that earlier repair after acute tears can yield better outcomes than delayed intervention [7, 15, 17].

We also order (often appropriately, but sometimes repetitively) a cascade of resource‐intensive interventions while ‘watching’ the tear: physiotherapy, repeated imaging, injections (including corticosteroids and biologics), and various adjunctive treatments. Some of these, while valuable, collectively become costly and time‐consuming. Despite the cost, there is still a need for salvage procedures when the tendon becomes irreparable (subacromial spacers, superior capsular reconstruction, tendon transfers or reverse shoulder arthroplasty) [11, 20]. In other fields, when the natural history is clearly unfavourable, escalation is often earlier and more decisive. Shoulder surgery remains unusually tolerant of delay despite the known trajectory of tissue degeneration. It is reasonable to ask why we continue to accept a macro‐invasive infrastructure for problems that, at least in their early stages, might be addressed more directly and less invasively. Multiple medical disciplines have shifted from large‐access surgery towards catheter‐based, image‐guided and micro‐invasive intervention. Orthopaedic shoulder surgery has advanced, but it has not yet undergone a comparable step change in delivery. This is not an argument against arthroscopy, but an argument against assuming it is the endpoint.

The percutaneous era is not theoretical: it is already here

Ultrasound‐guided interventions around the shoulder are now mainstream in many health systems. Expert consensus documents already frame image‐guided shoulder procedures as common clinical practice, while explicitly calling for more substantial evidence [21]. Beyond injections [12, 14, 19], ultrasound‐guided percutaneous procedures such as needling and lavage of calcific deposits [24], and capsular perforation/release techniques for adhesive capsulitis (sometimes described as a ‘pie‐crusting’ approach) [25] have been described; in addition, groups have reported ultrasound‐guided percutaneous long head of biceps tenotomy in clinical practice [10] and prospective feasibility work using a minimally invasive approach under continuous ultrasound monitoring [22]. Importantly, the literature also contains cautionary data: cadaveric evaluation has questioned reliability and highlighted iatrogenic risk when technique and instrumentation are not optimal [1, 16]. The message is not ‘everyone should do this tomorrow’. The message is that the direction of progression is clear: if surgeons do not lead the maturation of these techniques, others will.

A turf shift is plausible, and we have seen this movie before

Cardiovascular care offers a cautionary parallel: many high‐volume procedures have migrated from open surgery to catheter‐based, image‐guided practice led by cardiologists and interventional radiologists. We should not let the belief that we have permanently secured the surgical ownership of shoulder repair turn into complacency. Many clinicians who currently refer patients to orthopaedic surgeons—sports medicine physicians, rheumatologists and radiologists—have adopted musculoskeletal ultrasound early and at scale. In several countries, they teach ultrasound certification pathways and control access to formal credentials. Under growing pressure to provide efficient, office‐based care, it is easy to foresee these clinicians moving beyond diagnostic ultrasound and injections [5]. Particularly with interventional procedures, such as navigation, dedicated micro‐instruments and standardised workflows lower technical barriers. If that shift accelerates, the key question is not theoretical; it is practical: why would a busy gatekeeper refer a patient with an early or ‘small‐lesion’ problem to a surgeon? When a costly, anaesthesia‐dependent, recovery‐dependent operating‐room pathway can be avoided with a less invasive intervention in the same clinic. Referral patterns are shaped by access, efficiency and perceived value. The answer cannot rest on tradition or presumed ‘ownership’; it must be earned through leadership, evidence and superior outcomes. If shoulder surgeons do not master ultrasound, navigation and percutaneous treatment now, in the future, ‘small lesion’ patients may never reach the operating room. Not because surgery is unnecessary, but because the first‐line procedural solution will migrate upstream into clinics that already have the imaging tool, the patient access, and the economic motivation. What begins with rotator cuff disease may soon extend to instability—what is more straightforward than an ultrasound‐guided remplissage?—and eventually to cartilage procedures. And then the circle is complete.

## WHAT SHOULD SURGEONS DO NOW?

The appropriate response is not territorial conflict. It is leadership, competence, and evidence. First, shoulder surgeons should regard ultrasound literacy as foundational rather than optional. It should be embedded in fellowship training and continuing education, with clear standards and assessment. Second, surgeons must stop thinking ‘macro’ and deliberately start thinking ‘micro’. That shift in mindset should translate into active participation in the development and validation of micro‐invasive workflows. We need to prioritise safety, supported by dedicated instruments, navigation when appropriate, cadaveric training, stepwise credentialing and rigorous complication reporting. The negative reliability data in cadaveric settings are not a reason to ignore the field; it is a reason to professionalise it [16]. Third, we should reframe our therapeutic threshold for early, small tears. If the biological cost of time is real, then the most meaningful innovation may not be another arthroscopic suture configuration, but a safe, reproducible and early minimally invasive intervention before degeneration is established [7, 8, 26]. Finally, we must generate the evidence that health systems demand: comparative effectiveness, patient‐centred outcomes, cost‐utility and clear indications. Consensus already acknowledges that evidence for many image‐guided shoulder interventions remains limited, creating an excellent opportunity for surgical leadership of subsequent research [21].

## CONCLUSION

Arthroscopy changed our speciality. The next disruptive change may not be a better arthroscope, but a different paradigm: ultrasound‐enabled navigation, micro‐instruments, percutaneous repair strategies and earlier intervention. Our aims should focus on preserving biology rather than compensating for its loss. If orthopaedic surgeons lead this transition, patients benefit and the speciality advances. If we dismiss it as someone else's domain, we should not be surprised if, once again, we find ourselves trying to ‘catch up’ after the boat has already left.

## CONFLICT OF INTEREST STATEMENT

Alexandre Lädermann is a paid consultant for Arthrex, Stryker and Medacta. He received royalties from Stryker and Medacta. He is the (co‐)founder of FORE, Med4Cast and BeeMed. He owns stock options in Follow Health. He is a member of the board of the French Arthroscopic Society. Giuseppe Milano received grants from Medics, consultant fees from Arthrex, payment or honoraria for lectures, presentations, speakers bureaus, manuscript writing or educational events from Rejoin and Arthrex and support for attending meetings and/or travel from Medi and Exactech. He participates on a Data Safety Monitoring Board or Advisory Board from Arthrex, and he receives equipment, materials, drugs, medical writing, gifts or other services from FGP and Medacta. He is Deputy Chair of the Shoulder Committee of ISAKOS. Danilo Vukanic declares no conflicts of interest.

## ETHICS STATEMENT

The authors have nothing to report.
